# The Prevalence of Depression Associated with the Infection Status and Sexual Behaviors among Men Who Have Sex with Men in Shenzhen, China: A Cross-Sectional Study

**DOI:** 10.3390/ijerph17010127

**Published:** 2019-12-23

**Authors:** Xia An, Qunlu Sun, Fang Fang, Zhanhong Rao, Xiaowen Li, Yunhong Lv, Tong Li, Aihua Lin

**Affiliations:** 1 School of Public Health, Sun Yat-sen University, Guangzhou 510080, China; 2 Baoan District Center for Disease Control and Prevention, Shenzhen 518101, China; 3 The Affiliated Wuxi Mental Health Center with Nanjing Medical University, Wuxi 214151, China

**Keywords:** depression, men who have sex with men (MSM), HIV, Shenzhen, China

## Abstract

Objective: To study the prevalence of depression and related factors among men who have sex with men (MSM) in Shenzhen China. Methods: Using a cross-sectional design, convenient sampling was applied to recruit participants at the AIDS(Acquired Immune Deficiency Syndrome)voluntary counseling and testing (VCT) clinic and gay clubs in 2015, thereby collecting data on sociodemographics, serological information, sexual behaviotablers, and depression. Descriptive analyses were conducted to determine the distribution of the measured variables. A chi-square test was applied to test the association between different levels of factors and depression status, alongside a binary logistic regression for multivariate analysis of depression. Results: A total of 334 MSM completed the survey. Their mean age was 29.88 ± 7.56, and 35.6% had at least college education; 44.9% considered themselves to be homosexual, and 43.4% considered themselves bisexual. The median score of depression was 12, with 116 people (34.7%) depressed. A total of 267 took the serological test. Of these 267, 60 (22.5%) were reported HIV(Human Immunodeficiency Virus) positive, 33 (12.4%) were syphilis positive, and none were hepatitis C positive. The multiple logistic regression analysis showed that a lack of awareness of AIDS knowledge (OR(Odds Ratio): 2.636, 95% CI(confidence interval): 1.384–5.020), peer education (OR: 1.752, 95% CI: 1.055–2.190), and lack of heterosexuality (OR: 1.805, 95% CI: 1.080–3.018) increased the odds of depression. Conclusion: Raising awareness of AIDS and strengthening peer education can improve depression among men who have sex with men.

## 1. Introduction

A large number of studies worldwide have shown that men who have sex with men (MSM) have a poor mental health status compared to those with only heterosexual partners. MSM also face general mental health conditions, such as drug abuse, depression, anxiety, and suicidal intentions and behaviors [1,2,3,4,5,6,7]. The risk for depression and anxiety disorders has been shown to be at least 1.5 times higher among lesbian, gay, and bisexual people compared to the heterosexual population, and the lifetime prevalence of suicide attempt was 4.4 times higher among gay and bisexual men [8]. A study [9] in China found that all gay men accept homosexuality while 76.5% of heterosexuals hold the same view in an explicit attitude test. However, it may be stated that implicit beliefs in homosexual stereotypes were apparently found among heterosexuals. Due to the lack of legal protections for the rights of gay groups, homosexuals are facing many legal problems. “No legal marriage” and “no adoption rights” are two major deficiencies in the legal and other normative rights of homosexuals. Because of their lack of social identity and legal protection, as well as their sexual orientation and sexual behaviors [10], in mainland China, MSM remain at the edge of mainstream culture, suffering from tremendous psychological external pressures, and being prone to mental disorders, such as depression. The detection rate of depression in China was 29.1%–51.5% in a previous meta-analysis [11]; however, there is only one research paper [12] published in a Chinese journal that reported the depression status of MSM in Shenzhen up to the present. Understanding the burden of disease among groups at increased risk is very important for any national response to public health among the MSM population.

Individual-level risks for HIV acquisition among MSM have been well documented [3,13,14], including unprotected anal intercourse with insufficient behavioral safety awareness, a high frequency of male partners, and a high number of temporary or lifetime male partners. The epidemic of AIDS/HIV is still a global public health and social issue, especially in such high-risk groups. In 2018, 1.7 million new infections occurred worldwide, and the number of HIV-infected people reached 37.9 million [15]. In 2017, 25.5% of the new infections were caused by homosexual behavior in China [16]; this proportion accounted for 48.8% in Shenzhen city, and for the first time exceeded the proportion of heterosexual transmission by 44.0% in 2014. From January to October 2018, homosexual HIV transmission reached up to 51.9% among the 1803 new HIV-infected cases, as reported by the Shenzhen Municipal Health Commission [17]. As the first special economic zone in China and a window city for China’s reform and opening-up, the new immigrant city of Shenzhen (with a preponderance of floating populations and geographical features adjacent to Hong Kong) has produced a unique and relaxed cultural environment; consequently, Shenzhen city has gathered MSM from all over China. As we can see, intervening in homosexual HIV transmission has become the top priority for AIDS prevention and control in Shenzhen, China. Of particular concern is the mental health status of the MSM population, mainly for depression, with very few studies at the local level.

In this survey, we sought to understand the prevalence of depression and its associated factors, as well as the infection status and sexual behaviors pertinent to MSM in Shenzhen, China in order to provide a scientific basis for proposing a reasonable reference for psychological–behavioral intervention services among the MSM population and support the local national demonstration area for the comprehensive prevention and treatment of AIDS.

## 2. Materials and Methods

### 2.1. Setting and Participants

Using a cross-sectional epidemiology design, data were collected anonymously through self-filling questionnaires between March and December 2015 in Shenzhen: a city with large quantities of HIV-positive MSM in Guangdong, China. Convenient sampling was applied to recruit participants at the AIDS voluntary counseling and testing (VCT) clinic and from gay clubs (both conducted under the assistance of the Baoan District Center for Disease Control and Prevention (CDC)). A pre-survey was conducted in February 2015, with twelve active MSM recruited by insiders submitting their opinions on the contents of the survey to improve the rationality of the questionnaire and the acceptability of the target population. There were two ways to reach potential respondents at the two sites used for recruitment. The doctors at the VCT clinic informed the MSM who were actively seeking counseling and testing services about recommended, qualified people who could come over and test them. In addition, based on cooperation with and assistance from gay social organizations, questionnaires and blood samples were taken at gay social activities, and the blood was brought back for testing by staff. Each participant received CNY 50 as compensation for their time spent on the survey. All research activities were managed by experienced research assistants in the MSM and HIV prevention fields. Participants were briefed about the study’s purpose and then provided written informed consent before completing the survey questionnaire. The participant self-administered the questionnaire using a pen and paper form, with an explanation given by the research assistant if the participant had any difficulty with any question or item, as well as to check for completeness and consistency of the data while the participant was still present.

Individuals were eligible for the study if they were male, 18 years or older, could provide informed consent for the study, and indicated that they had sex with another male prior to data collection. Those who had severe cognitive impairment or communication disorders were excluded. A total of 466 eligible participants were approached, of whom 363 (a response rate of 78%) participated in the survey. Of the 363 questionnaires collected, 29 were unqualified due to a lack of key information (a qualified rate of 92%), resulting in a total of 334 participants. Ethics approval was obtained from the ethics committee of Sun Yat-sen University.

### 2.2. Measurements

General demographic characteristics include age, nationality, census register, education level, occupation, marital status, monthly income, etc., were collected through self-filling questionnaires.

Serological information included the routine items of VCT clinic and outreach services for high-risk populations, including antibody testing for HIV, syphilis, and hepatitis C. In strict accordance with the “National AIDS Testing Technical Specifications” (revision of 2009), the Baoan district CDC used an enzyme-linked immunosorbent assay (ELISA) to screen HIV antibodies, and western blotting (WB) for confirmation. Syphilis antibody screening was performed by a rapid plasma reagin (RPR) test and a *Treponema pallidum* particle assay (TPPA) for confirmation, in cooperation with the Baoan District Chronic Disease Prevention and Treatment Institute. The hepatitis C antibody was detected by ELISA.

AIDS prevention and treatment included eight questions on AIDS knowledge adopted by the national sentinel surveillance [18] (the evaluation index contains six questions or more, with the right answer to be determined). Prevention and treatment also included access to AIDS knowledge and the utilization of AIDS-related intervention services, including access to AIDS intervention services regardless of whether they had tested for HIV, peer education, and free condom/brochure distribution.

Sexual behaviors included self-reported sexual orientation and whether they had revealed it to their families, their age at their first sexual encounter, and the gender of their first sexual partner, their number of sexual partners in the last six months, their role in anal sex and oral sex, heterosexual behavior, and condom use.

Depression: The Center for Epidemiological Studies Depression Scale (CES-D) [19] was used to measure the occurrence of depressive symptoms. This scale has 20 items according to the frequency of the corresponding situation or feeling during the last week: Less than one day is “no or basically no”, 1–2 days is “rare”, 3–4 days is “often”, and 5–7 days is “almost always “. The scores were recorded as 0, 1, 2, and 3 points, respectively. The higher the total score of the 20 entries, the higher the level of depression. Usually, 16 points is used as the demarcation point for depression, and this criterion was also followed in our study. The Cronbach’s coefficient of the internal consistency test for the scale in this study was 0.88.

### 2.3. Statistical Methods

The database was built using EpiData version 3.1 (EpiData Association, Odense Denmark) with the questionnaire given double entries for consistency. Descriptive analyses were conducted to determine the distribution of the demographic factors and other measured variables. A chi-square test was applied to test the association between the different levels of factors and depression status, as well as a binary logistic regression for multivariate analysis of depression. According to an empirical estimate of 10 times the covariates, the existing sample size meets these conditions. All statistical analyses were performed with SPSS version 21.0 (IBM Corp, Armonk, NY, USA), and a two-tailed *p* < 0.05 was considered to be statistically significant.

## 3. Results

### 3.1. Demographic and Serological Variables

A total of 334 MSM completed the survey, including 142 (42.5%) samples from VCT and 192 (57.5%) from clubs. The mean age was 29.88 (SD = 7.56) years, among whom 68.3% were single and 25.4% were married; also, 82.3% were not registered in the Guangdong census. A total of 267 participants among them took the serological test; 60 (22.5%) were reported to be HIV positive, 33 (12.4%) were syphilis positive, and none were hepatitis C positive. Other sociodemographic characteristics, such as the degree of education, personal income, etc., are detailed in Table 1.

### 3.2. AIDS Prevention and Treatment

Of the eight questions about AIDS knowledge adopted by the national sentinel surveillance, 267 MSM gave the correct answers to six or more questions, and the total awareness rate was 79.9%. The details of the participants’ HIV/AIDS knowledge are shown in Table 2. For other HIV/AIDS service utilization, 83.8% knew about VCT services, only 33.4% understood the Four Free and One Care policy, 53.3% tested for HIV over the past year, 44.6% received condoms/brochures, and 52.7% had experienced peer education. The intervention service that the subjects received the most was online intervention, accounting for 82.6%, which was also the most preferred method of communication (67.6%). Other intervention services received were advertisement columns (43.4%), television (41.9%), newspapers/books (38.9%), and doctors (22.8%).

### 3.3. Sexual Behaviors

Among the respondents, 150 (44.9%) considered themselves to be homosexual, 145 (43.4%) bisexual, and 39 (11.7%) uncertain. A total of 231 respondents (69.2%) had revealed their sexual orientation to their families. The age of the participants at their first sexual encounter ranged from 12 to 35 years old (average 21.2 ± 3.7), and 182 of the participants (54.5%) had their first sexual encounter with a homosexual. The number of sexual partners in the last six months ranged from 0 to 50, mainly concentrated around 1–6 (average 2.9 ± 3.9), and 224 participants (67.1%) had sexual intercourse with two or more partners (multiple partners) in the past six months. Details are shown in Table 3.

### 3.4. Depression

The scores for depression ranged from 0 to 48, the median was 12.00, and the interquartile range was 15.75; the score was non-normally distributed, so these statistics were used. Further, 116 (34.7%) of the participants were depressed, and 218 were not.

### 3.5. Univariate Analysis of Depression

For MSM with different sociodemographic characteristics, HIV/AIDS knowledge, different sexual behaviors, and rate of depression was determined by a chi-square test. Social demographics were not found to be related to depressive symptoms. There was no statistically significant difference in depression rates between the serological results (HIV testing, *p* = 0.369. syphilis testing, *p* = 0.175, hepatitis C testing, *p* = 0.348), and details are shown in Table 4. If we leave out those who refused testing, there was still no statistically significant difference in the depression rates between serological results (HIV testing, *p*= 0.295. syphilis testing, *p* = 0.109). The total awareness of HIV/AIDS knowledge (*p* = 0.012) was statistically significant for the depression of MSM. For single questions, the awareness of “Can proper use of condoms reduce the spread of AIDS?”(*p* < 0.001) and “Does having sex with only sexual partner reduce the spread of AIDS?”(*p* = 0.009) were statistically significant while the other six questions were not; the gender of their first sexual partner, sexual behavior during anal/oral sex, having anal sex in the last six months, and heterosexual behavior were statistically significant (*p* < 0.05) determinants for the depression of MSM; details are shown in Table 3.

### 3.6. Multivariate Analysis of Depression

Variables in univariate analysis with a *p*-value lower than 0.2 were used as independent variables, while depression was the dependent variable (depression = 1, non-depression = 0). Using the forward LR (likelihood-ratio test) method, including the standard of 0.10 and excluding the standard of 0.05, a dummy variable was set for the categorical variables. To perform a multivariate logistic regression analysis, the results showed that awareness of AIDS knowledge, peer education, and sexual behavior with women were influencing factors of depression for MSM. AIDS knowledge, experiences with peer education, and heterosexual behaviors were relatively protective factors, as shown in Table 5.

## 4. Discussion

According to our knowledge, this is a rare type of study collecting information on the prevalence of depressive symptoms and related factors among MSM in Shenzhen, China. In this study, 34.7% of MSM among the 334 respondents had depression symptoms. Compared to previous findings, the results of the depression rate were consistent with studies in northeast China and Jiangsu province (respectively, 33.1% and 26.8% [20,21]) and higher than a study in Zhengzhou [22], which found only 20.3% to suffer from depression. One reason for this discrepancy may be that different measurement scales have different evaluation purposes and methods, leading to differences between the research results. The CES-D scale [23] is usually used as a screening tool in clinical practice and has high sensitivity, which may have led to the higher depression prevalence found in our study. In studies carried in Shanghai and west China, 52.1% and 50.8% had depression symptoms [2,24], which is higher than the results in our study; despite adopting the same depression scale, these discrepancies might be due to differences in culture and HIV infection status.

The results of the multivariate analysis showed that a lack of awareness of AIDS knowledge (OR: 2.636, 95% CI: 1.384–5.020), peer education (OR: 1.752, 95% CI: 1.055–2.190) increased the odds of depression, while more peer educators with a high informational support profile was related to higher self-efficacy, social support, and behavior management [25]. In addition, those not identifying as heterosexual seemed to more readily become depressed (OR: 1.805, 95% CI: 1.080–3.018), in contrast with another finding [2]; the prevalence of depression was 35.18% among men who have sex with men only and 50.86% among men who have sex with men and women, which may be related to marital status. This result seems inconclusive with regard to the relationship between depression and the gender of one’s sex partners.

Serological results were not associated with depression in the univariate analysis in our study. For the individuals who took the serological test and had a positive result in the investigation, these serological results were not clear while filling out the questionnaires. Previous studies [26,27,28,29] paid much attention to the quality of life among people living with HIV (PLHIV). The frequency of major depressive disorder was nearly two times higher among HIV-positive subjects than among HIV-negative comparison subjects [30]. The psychological status of the HIV-positive population should be continuously examined in follow-up interventions. Rates of depression do not appear to be related to sexual orientation, which is consistent with previous research [30], but others have found that gay/lesbian participants reported more acute mental health symptoms than did heterosexual people, and their general mental health was also poorer [31]. Using a 36-Item Short-Form Health Survey scale may lead to inconsistent results. Moreover, the *p*-value is very close to the boundary in our study, so we cannot determine a certain conclusion.

For the results of the serological test, 22.5% (60/267) were HIV positive (higher than the Shenzhen sentinel monitoring data from 2012 [32]), and 12.4% (33/267) were syphilis positive, including 4.11% (11/267) who were positive for both HIV and syphilis. Other evidence [33] showed that HIV and syphilis incidence rates were 7.83 and 11.11 per 100 person-years, respectively. However, the positive rate in this study may be higher or lower than the actual situation for the following two reasons. Most of the participants were afraid of infection because of their high-risk behaviors or related symptoms, so they actively sought testing through the VCT; on the other hand, 96.7% of the respondents said they were willing to undergo blood tests, while only 79.9% actually received the test, so it is reasonable to speculate that investigators who refuse to be tested are more likely to be positive, as there were no previous test results in the questionnaire. The place network is an activity network that is mostly accessible to people outside the MSM community. In the course of this study, more people were recruited by the clubs than actively sought services through the VCT. Thus, we must consider that intervention services through the place network may provide the possibility of determining more potential sexual partners in clubs while offering AIDS intervention services to reduce risky sexual behaviors.

There are potential limitations to this study. Firstly, although the research process followed strict quality control, it is inevitable that an investigation of sensitive information, such as sexual orientation, sexual partners, sexual behaviors, and other relatively personal information, may be concealed in the self-report to some extent, thereby affecting the results of analyzing the influencing factors. Second, we were not able to use a random sampling scheme, so there may be a lack of representation for all sub-populations among Shenzhen-based MSM. Consequently, our findings may not be generalizable to other regions of China. However, most studies on MSM populations rely on improbability/approximate probability methods for relatively invisible groups, making it difficult to obtain good results through traditional sampling methods [34]. We used a cross-sectional methodology lacking evidence strong enough to determine causal inference. Thirdly, data on serological results prior to the study were lacking, so we cannot confirm the difference of depression among PLHIV and HIV-negative MSM in the area. Fourthly, measures of marginalization or discrimination, which are likely to have accounted for the association between depression and other factors, were unfortunately not covered in this survey but will be considered for further measurements in future research.

## 5. Conclusions

We provide evidence of depression and its influencing factors, as well as the serological results of MSM in Shenzhen, China. We believe that awareness of AIDs and peer education help with depression so that relevant measures can be strengthened in practical work. People with MSM may have a relatively closed social state or self-identity; more efforts are needed to improve depression and possibly to improve traditional social identity to eliminate discrimination and homophobia in mainland China.

## Figures and Tables

**Table 1:** Social demographics of the participants (*n* = 334).

Characteristics	*n*/(%)
*Age group, year*	
<30	188 (56.3)
30~	101 (30.2)
40~	45 (13.5)
*Degree of education*	
Middle school and below	92 (27.6)
High school or technical school	123 (36.8)
College degree or above	119 (35.6)
*Marital status*	
Single	228 (68.3)
Married	85 (25.4)
Divorced or widowed	21 (6.3)
*Personal monthly income, CNY*	
<3000	82 (24.5)
3000~	140 (41.9)
5000~	81 (24.3)
8000~	31 (9.3)
*Shared living situation*	
Alone	142 (42.5)
With friends, colleagues, strangers	94 (28.1)
With a sexual partner	36 (10.8)
With families	62 (18.6)
*Willingness to get married*	
Yes	148 (44.3)
No	101 (30.2)
Uncertain	85 (25.5)
*The only child*	
Yes	148 (44.3)
No	186 (55.7)
*Time living in Shenzhen*	
Less than 1 year	66 (19.8)
More than 1 year	268 (80.2)
*Census register*	
Guangdong	59 (17.7)
Others	275 (82.3)
*Nationality*	
Han	301 (90.1)
Others	33 (9.9)

**Table 2:** Awareness rate of AIDS knowledge.

Questions	Number Aware	Awareness Rate/%
Can an HIV-infected person be noticed based on external factors?	216	64.7
Will mosquito bites spread AIDS?	210	62.9
Is it possible to get infected by having a meal together with an HIV-infected person or AIDS patient?	276	82.6
Can HIV be transferred when blood with HIV is transfused?	319	95.5
Is it possible to get AIDS by sharing a syringe with people living with HIV?	318	95.2
Is it possible for children born to HIV-infected women to have AIDS?	303	90.7
Can the proper use of condoms reduce the risk of HIV infection?	299	89.5
Does having sex with only one sexual partner reduce the risk of HIV infection?	268	80.2
Total awareness (defined by knowing six questions or more)	267	79.9

**Table 3:** Descriptions of sexual behaviors and services used.

Variables	n/(%)	Depressed n/(%)	Not Depressed n/(%)	**$\chi^{2}$**	*p*
*Sexual orientation*				5.784	0.055
Homosexual	150 (44.9)	60 (40.0)	90 (60.0)		
Bisexual	145 (43.4)	40 (27.6)	105 (72.4)		
Uncertain	39 (11.7)	16 (41.0)	23 (59.0)		
*Ever received condom distribution*				3.209	0.073
Yes	149 (44.6)	44 (59.5)	105 (70.5)		
No	85 (25.4)	72 (84.7)	13 (15.3)		
*Peer education*				2.690	0.101
Yes	176 (52.7)	54 (30.7)	122 (69.3)		
No	158 (47.3)	62 (39.2)	96 (60.8)		
*Knowledge of VCT*				1.756	0.185
Yes	280 (83.8)	93 (33.2)	187 (66.8)		
No	54 (16.2)	23 (42.6)	31 (57.4)		
*Ever tested*				1.798	0.180
Yes	178 (53.3)	56 (31.5)	122 (68.5)		
No	156 (46.7)	60 (38.5)	96 (64.5)		
*Gender of first sexual partner*				6.201	0.013
Male	182 (54.5)	74 (40.7)	108 (59.3)		
Female	152 (45.5)	42 (27.6)	110 (72.4)		
*Relationship with first sexual partner*				5.576	0.062
Elder	21 (6.3)	10 (47.6)	11 (52.4)		
Colleagues and friends	198 (59.3)	59 (29.8)	139 (70.2)		
Netizens	115 (34.4)	47 (40.9)	68 (59.1)		
*More than one sexual partner*				3.103	0.078
Yes	224 (67.1)	85 (37.9)	139 (62.1)		
No	110 (32.9)	31 (28.2)	79 (71.8)		
*Anal sex role*				5.471	0.065
1 *	91 (29.1)	24 (26.4)	67 (73.6)		
0 *	61 (19.5)	23 (37.7)	38 (62.3)		
Both	161 (51.4)	66 (41.0)	95 (59.0)		
*Sexual behavior*				8.300	0.016
Anal sex only	41 (12.3)	9 (22.0)	32 (78.0)		
Oral sex only	21 (6.3)	3 (14.3)	18 (85.7)		
Both	272 (81.4)	104 (38.2)	168 (61.8)		
*Anal sex in the last six months*				4.132	0.042
Yes	313 (93.7)	113 (36.1)	200 (63.9)		
No	21 (6.3)	3 (14.3)	18 (85.7)		
*Frequency of condom use in anal sex*				1.776	0.620
Every time	145 (46.3)	50 (34.5)	95 (65.5)		
Most of the time	131 (41.9)	46 (35.1)	85 (64.9)		
Occasionally	26 (8.3)	12 (46.2)	14 (53.8)		
Never	11 (3.5)	5 (45.5)	6 (54.5)		
*Oral sex in the last six months*				3.367	0.067
Yes	293 (87.7)	107 (36.5)	186 (63.5)		
No	41 (12.3)	9 (22.0)	32 (78.0)		
*Frequency of condom use in oral sex*				1.993	0.574
Every time	21 (7.2)	9 (42.9)	12 (57.1)		
Most of the time	14 (4.8)	6 (42.9)	8 (57.1)		
Occasionally	52 (17.7)	15 (28.8)	37 (71.2)		
Never	206 (70.3)	77 (37.4)	129 (62.6)		
*Heterosexual behavior*				5.584	0.018
Yes	210 (62.9)	63 (30.0)	147 (70.0)		
No	124 (37.1)	53 (42.7)	71 (57.3)		
*Frequency of condom use in heterosexual intercourse*				5.042	0.169
Every time	79 (37.6)	26 (32.9)	53 (67.1)		
Most of the time	45 (21.4)	18 (40.0)	27 (60.0)		
Occasionally	33 (15.7)	7 (21.2)	26 (78.8)		
Never	53 (25.3)	12 (22.6)	41 (77.4)		

VCT—voluntary counseling and testing; * note: 1—the penetrative role in sexual intercourse (in particular refers to “anal” and “oral sex”), 0—the receptive role in sexual intercourse (in particular refers to “anal” and “oral sex”).

**Table 4:** Descriptions of serological results.

Serological Variables	*n*/(%)	Depressed *n*/(%)	Not depressed *n*/(%)	$\chi^{2}$	*p*
*HIV testing*				1.944	0.369
Positive	60 (18.0)	25 (41.7)	35 (58.3)		
Negative	207 (62.0)	71 (34.3)	136 (65.7)		
Refused testing	67 (20.0)	20 (29.9)	47 (70.1)		
*Syphilis testing*				3.488	0.175
Positive	33 (9.9)	16 (48.5)	17 (51.5)		
Negative	234 (70.1)	80(34.2)	154 (65.8)		
Refused testing	67 (20.0)	20 (29.9)	47 (70.1)		
*Hepatitis C testing*				0.880	0.348
Negative	267 (80.0)	96 (36.0)	171 (64.0)		
Refused testing	67 (20.0)	20 (29.9)	47 (70.1)		

**Table 5:** A multivariate logistic regression analysis of the influencing factors of depression.

Influencing Factors	*β*	Wald $\chi^{2}$	*p*	OR (95% CI)
*Awareness rate of AIDS knowledge*				
Yes				1.000
No	0.069	8.694	0.003	2.636 (1.384–5.020)
*Peer education*				
Yes				1.000
No	0.561	4.691	0.030	1.752 (1.055–2.190)
*Heterosexual behavior*				
Yes				1.000
No	0.591	5.073	0.024	1.805 (1.080–3.018)
